# Recurrent Severe Burns Due to Cannabinoid Hyperemesis Syndrome

**DOI:** 10.7759/cureus.34552

**Published:** 2023-02-02

**Authors:** Eugene Osagie, Omar Mirza

**Affiliations:** 1 Psychiatry, Harlem Hospital Center, New York, USA; 2 Psychiatry, Columbia University College of Physicians and Surgeons, Harlem Hospital Center, New York, USA

**Keywords:** vomiting, nausea, marijuana, cannabis, cannabinoid hyperemesis syndrome

## Abstract

Cannabinoid hyperemesis syndrome (CHS) is one of the health outcomes of cannabis use that is showing an increase in the number of reported cases since it first emerged in the medical literature. It is a condition that is now frequently seen by many specialists, including consultation-liaison psychiatrists. CHS is a diagnosis of exclusion that is characterized by the presence of a prolonged history of daily cannabis use, cyclic episodes of nausea and vomiting, and frequent compulsive hot baths.

It will be fairly argued that with the surge in the number of marijuana users and frequency of use since the legalization of marijuana in the United States, a proportionate rise in CHS cases is expected over time.

In this case report, we present a unique case of a 36-year-old female diagnosed with CHS, and the compensatory behavior of compulsive hot baths led to repeated episodes of severe burns, sepsis, and intensive care unit (ICU) hospitalizations. To the authors' knowledge, this is the first published case of severe burns and sepsis as complications of cannabinoid hyperemesis syndrome.

## Introduction

Cannabis is one the most widely used substance in the United States after alcohol and tobacco; about 19% of Americans over the age of 18 use marijuana at least once over a 12-month period [1]. Recreational cannabis use has traversed several policy landscapes, ranging from criminalization to decriminalization to legalization. Cannabis use may be overlooked during clinical assessments due to changes in perception by clinicians brought on by policy, and this can impact the risks associated with cannabis use [2]. It is imperative to continue to highlight the known adverse effects of cannabis use.

Cannabis hyperemesis syndrome (CHS) is a condition that is characterized by a long history of daily cannabis use, cyclic episodes of nausea and vomiting, and frequent hot bathing or showering [3].

CHS is frequently seen in emergency rooms (ER) among patients with a history of cannabis use. The consultation-liaison psychiatrists are frequently involved in the care of these patients due to nausea and vomiting that lead to admission with no identifiable cause. One of the hallmark symptoms that distinguishes this condition is the narrative of hot showers for symptomatic relief. In a literature review, compulsive bathing behavior was reported in 29 out of 31 previously reported cases of CHS [4].

In this case report, we present a case of CHS that is unique behavior in that the compensatory penchant for hot showers led to repeated episodes of severe burns and raise the awareness of potential dangers that can arise from this behavior.

To our knowledge, this type of complication has not been previously reported in the literature. Our database search includes PubMed, Scopus, CINAHL, PsychINFO, and ScienceDirect.

## Case presentation

Our patient is a 36-year-old female previously diagnosed with psychogenic seizures, bipolar disorder, borderline personality disorder, substance use disorder (cocaine and cannabis), gastritis, and irritable bowel syndrome who was brought in by ambulance to the emergency department following burns to her torso sustained in the bathtub. Earlier in the day, she started experiencing abdominal discomfort and intractable non-bilious, non-bloody vomiting and decided to spend time in the bathtub to help alleviate the distress caused by repeated vomiting and nausea as she had done in the past.

**Figure 1:**
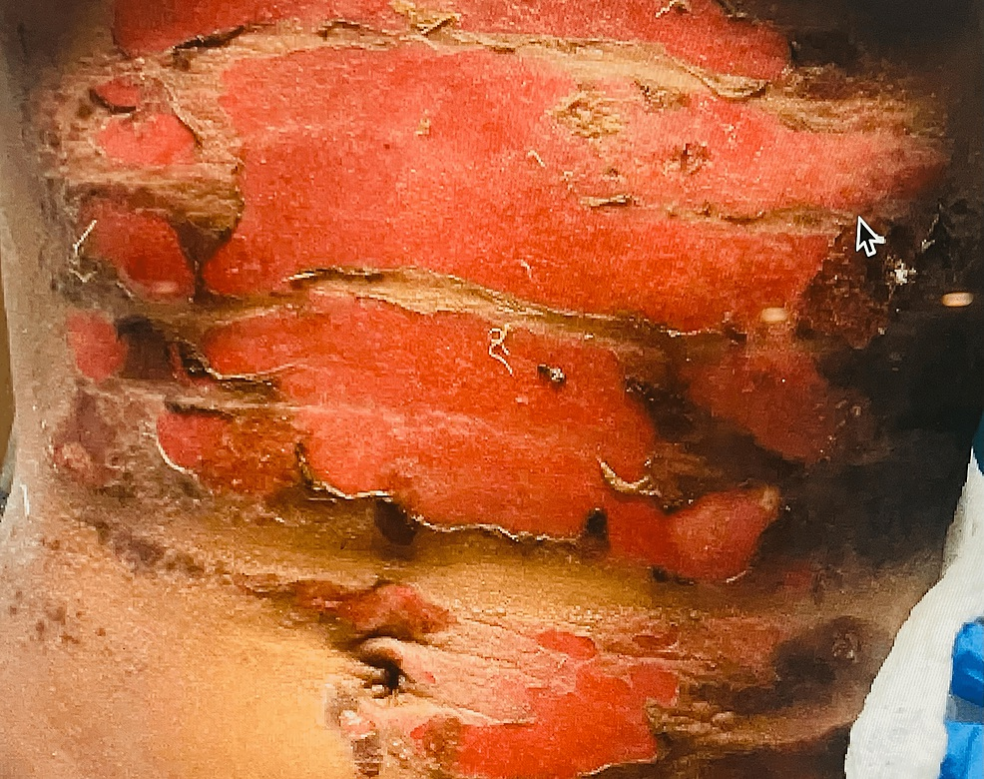
Anterior chest burn including breasts.

**Figure 2:**
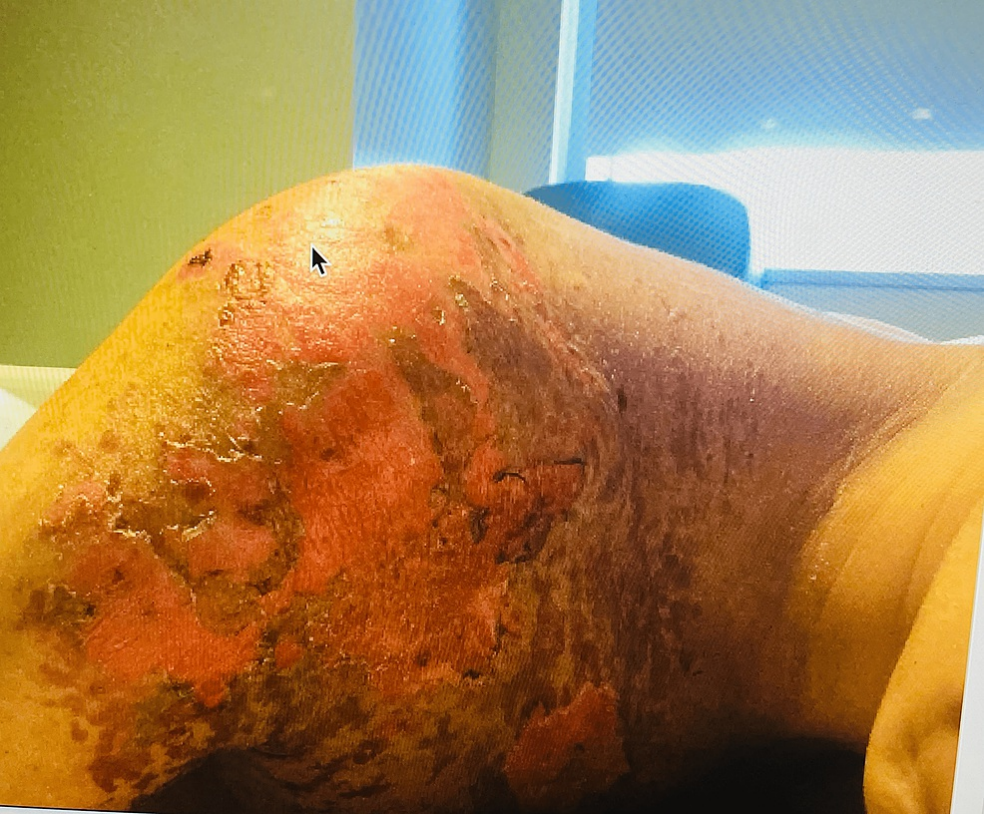
Right shoulder burn.

Physical examination findings included blood pressure 114/85, heart rate 98 bpm, oxygen saturation 99%, and temperature 97.2 F. She sustained second-degree burns involving up to 19% of the total body surface, mostly affecting her anterior chest, including the breasts (Figure 1), posterior thorax, and right shoulder (Figure 2). Laboratory findings include white blood cell count (WBC) 18.60 × 10^3^/mcL, sodium (Na) 130 mmol/L, creatinine (Cr) 2.3 mg/dL, blood urea nitrogen (BUN) 46 mg/dL, chloride (Cl) 73 mmol/L, anion gap (AG) 30 mEq/L, and alkaline phosphatase 111 U/L. Urine toxicology was positive for tetrahydrocannabinol (THC). She was admitted to the burn intensive care unit (ICU), where she received wound treatment, fluid and electrolyte replacement, and antibiotic therapy.

A review of her medical records showed that she had similar experiences of recurrent nausea, vomiting, and abdominal pain of approximately five years' duration that necessitated multiple emergency room (ER) visits and hospitalizations. She had two previous hospitalizations for severe burns. She received extensive workup for her condition, including laboratory tests, multiple computed tomography (CT) of the abdomen, kidney ultrasound, and esophagogastroduodenoscopy. Except for electrolyte derangements, and urine toxicology positive for tetrahydrocannabinol and cocaine, no acute pathology possibly causing her symptoms were identified. Electrolyte abnormalities were likely secondary to episodes of protracted vomiting episodes. Several medications, including famotidine, pantoprazole, and ondansetron, were prescribed during her hospitalizations.

The patient has been a daily cannabis user since the age of fourteen and has graduated up to using seven blunts per day. Her longest period without using cannabis since her first use was for four months while she was hospitalized for a left tibia fracture, during which time her symptoms dissipated but remerged when she resumed usage after she was discharged from the hospital.

Three years after the onset of her recurrent nausea and vomiting, she recognized her penchant for hot water baths to alleviate her pain. She started spending prolonged periods of time in the bathtub, and she gradually was able to tolerate water at hotter temperatures. This led to two previous hospitalizations for a similar degree of burns. In both cases, she self-activated the ambulance after she noticed that she had sustained bodily burns.

A multidisciplinary treatment approach, including medicine, general surgery, plastic surgery, oral surgery, and psychiatry, was applied. A comprehensive psychiatric evaluation found her to be psychiatrically stable with no signs of decompensated bipolar disorder. There were no reports of emotional dysregulation preceding the burn incident suggesting that the burn might be due to self-injurious behavior as seen in borderline personality disorder.

She was assessed to have a severe form of cannabinoid hyperemesis syndrome that is complicated by recurrent and potentially fatal burns from compulsive hot baths. She was stabilized and provided education about her illness, counseled on cannabis cessation, and offered a referral to an outpatient substance use treatment program.

## Discussion

This prevalence of cannabis use is predicted to see a significant rise following the legalization of cannabis in the United States in 2016 [5]. Cannabis hyperemesis syndrome (CHS) first emerged in the medical literature in 2004. It is a condition that is characterized by a long history of daily cannabis use, cyclic episodes of nausea and vomiting, and frequent hot bathing or showering [3].

The actual pathogenesis of cannabis hyperemesis syndrome is unknown; it seems paradoxical considering that cannabinoids are known anti-emetics that have been used in the treatment of conditions such as chemotherapy-induced vomiting and cyclical vomiting syndrome. The current hypothesis is that chronic overstimulation of endocannabinoid receptors, alters the body’s intrinsic control of nausea and vomiting [6]. There is the involvement of a neuroendocrine process that includes activation of the area postrema by noxious stimuli and stimulation of the vagus nerve leading to retro-peristaltic actions that culminate in the expulsion of the vomitus. The CB1 and CB2 receptors found in several neurologic and endocrinologic pathways and gastrointestinal tracts can also directly influence the process when stimulated [6].

The proposed clinical course of cannabinoid hyperemesis syndrome is divided into three phases: prodromal, hyperemetic, and recovery phases [3]. The hyperemetic phase is characterized by episodes of severe and persistent nausea and vomiting that can be overwhelming and incapacitating, and a resultant learned behavior of compulsive hot baths that provide temporary relief [3]. Soriano-Co et al., found that among eight patients with CHS, the average age of onset was 32.4 ± 4.1 years old, and the mean interval between the start of cannabis use and the onset of illness was 19.0 ± 3.7 years [7]. All the patients studied had at least three episodes of vomiting/day, and the mean total bathing time = 5.0 ± 5.1 h/day, mean of 7.1 ± 4.37 [7].

The diagnosis of this condition may be delayed or missed due to the broad differential diagnosis of abdominal pain, nausea and vomiting and unexplored cannabis use as part of a broad differential diagnosis in patients presenting with these symptoms [3]. Treatment involves supportive therapy with fluid resuscitation and anti-emetic medications, including benzodiazepines, antipsychotics, proton pump inhibitors, and cessation of cannabis use [6,8].

The compulsive bathing seen in this syndrome is a learned behavior that is hypothesized to be a pathognomonic feature because it has not been associated with any other vomiting disorder. It provides temperature-dependent symptomatic relief, with hotter water yielding more effective relief [9]. The need for frequent hot baths is typically not present at the onset of nausea and vomiting, but once it appears, it rapidly becomes a compulsion [9]. The pathophysiology of the compulsive hot baths seen in CHS is not well-understood but it is postulated that prolonged cannabis use may cause impairment in thermoregulation overtime [9]. It is also proposed that compulsive hot water bathing relieves nausea and cyclic vomiting through the hypothermic effects of THC or activation of CB1 receptors in the hypothalamus [4]. The anxiolytic effect of cannabinoids can lead to a reduction of psychological stress from persistent nausea and vomiting [4].

Remarkably, there is a huge difficulty in the treatment of CHS as the patient will not respond to traditional anti-emetic medications such as ondansetron and metoclopramide. The historical evidence of partial or complete relief from hot baths has led to a discussion about the potential benefit of heat therapy in management [6].

The mainstay of treatment is cessation of cannabinoid use. Soriano-Co et al. found that four out of five patients who successfully ceased cannabinoid use recovered from the syndrome, while the other three patients who persisted in their cannabis use, continued to have this syndrome [7]. Among those four who recovered, one patient had a recurrence of vomiting and compulsive bathing with a cannabis relapse [7].

Complications of CHS include electrolyte abnormalities, fluid imbalance, dehydration, and nutritional deficiencies. Uncontrollable and protracted vomiting can lead to aspiration pneumonitis or pneumonia, Mallory-Weiss tear, and Boerhaave's syndrome [6].

Our patient's demographic data, length of cannabis use, and presenting symptoms except burns are similar to those reported by Soriano-Co et al [7]. Over time, she developed a higher tolerance for hot water and started needing hotter water to provide relief for her gastrointestinal symptoms. This resulted in a total of three episodes of severe burns, requiring hospitalizations, including ICU stay and with complications of sepsis, rhabdomyolysis and high anion gap metabolic acidosis. The complications with potentially fatal burns from compulsive bathing seen in CHS have not previously been reported.

## Conclusions

It is expected that with the legalization of cannabis, the number of users and frequency of use will increase. Clinicians should be aware of the potential for an increased incidence of CHS and its complications. It is important to obtain a detailed history of cannabis use in any patient presenting with nausea and vomiting to the emergency room. Patients should be asked specifically about compulsive hot showers, and patients reporting this compensatory behavior should be educated about the risk of developing hot water tolerance and severe burns. Prompt diagnosis, treatment, and patient education can reduce the number of emergency room visits, hospitalizations, and potentially deleterious complications of CHS, including severe burns as described in this case report. Based on our search, this is the first report of severe burns as a complication of CHS. 
